# The complete chloroplast genome of *Catalpa speciosa* (Warder) Engelmann (Bignoniaceae)

**DOI:** 10.1080/23802359.2020.1765213

**Published:** 2020-05-14

**Authors:** Qing-guo Ma, Jian-guo Zhang, Jun-pei Zhang

**Affiliations:** State Key Laboratory of Tree Genetics and Breeding, Key Laboratory of Tree Breeding and Cultivation of National Forestry and Grassland Administration, Research Institute of Forestry, Chinese Academy of Forestry, Beijing, China

**Keywords:** *Catalpa speciosa*;· chloroplast genome, sequencing, phylogenetic analysis

## Abstract

*Catalpa speciosa* (Warder) Engelm. is a precious *Catalpa* tree widely used as a large ornamental shade tree. In this study, the complete chloroplast genome was assembled from Illumina sequencing data. The completed plastome was 158,239 bp in length, including a large single copy (LSC) region of 85,036 bp, a small single copy (SSC) region of 12,639 bp, and a pair of inverted repeat (IR) regions of 30,282 bp. The overall GC content of the genome was 38.09%, and the corresponding values of the LSC, SSC and IR regions were 36.44%, 33.57%, and 41.35%, respectively. A total of 129 genes were annotated, including 83 protein-coding genes, 38 tRNA genes, and 8 rRNA genes. Phylogenetic analysis was carried out based on complete plastome sequences of *C. speciose* and 11 other species from Bignoniaceae family. The newly characterized complete chloroplast genome will provide essential data for further studies of *C. speciosa*.

*Catalpa speciosa* (Warder) Engelmann, belonging to the genus *Catalpa* (Bignoniaceae), is a precious deciduous broad-leaved tree species native to central and eastern United States and cultivated widely in China. It has been used as a large ornamental shade tree and widely planted in urban areas as a street and lawn tree. Its leaves were used to remove Pb(II) from aqueous solutions (Zolgharnein et al. 2015). *Catalpa speciosa* bears important research value, but fundamental genetic information remains less. In the present study, we obtained the complete chloroplast genome sequence of *C. speciosa* based on Illumina sequencing data, which will provide useful reference for further studies of this species.

Leaves of *C. speciosa* were collected from the eastern campus of Hebei Agricultural University, Hebei Province (38°50′55″N, 115°28′39″E). The specimen was stored at Key Laboratory of Tree Breeding and Cultivation of National Forestry and Grassland Administration with the specimen code of HJShb1901. The total genomic DNA was extracted using modified CTAB protocol described by Wang et al. (2015) and sequenced using the Illumina Hiseq X Ten (Illumina, CA, USA). The raw reads were filtered using Cutadapt version 1.9.1 (Martin 2011). Clean reads were first aligned to *Tecomaria capensis* (NC_037462) and then assembled using Velvet v1.2.10 (Zerbino and Birney 2008) and NOVOPlasty v2.7.2 (Dierckxsens et al. 2016). The genome was annotated using GeSeq (Tillich et al. 2017) and tRNAscan-SE v2.0 (Lowe and Chan 2016). The annotated genomic sequence was then deposited into GenBank with accession number MT319818.

The chloroplast genome of *C. speciosa* was 158,239 bp in length and consisted of a large single copy (LSC) region of 85,036 bp, a small single-copy (SSC) region of 12,639 bp, and 2 inverted repeat (IR) regions of 30,282 bp each. The chloroplast genome encoded 129 genes, including 83 protein-coding genes, 38 tRNA genes and 8 rRNA genes. The overall GC content was 38.09% and GC content in the LSC, SSC and IR regions were 36.44%, 33.57%, and 41.35%, respectively.

The phylogeny of *C. speciosa* plus 11 other published chloroplast genomes of Bignoniaceae (*Adenocalymma trifoliatum* (NC_037459.1), *A. cristicalyx* (NC_036498.1), *Amphilophium dolichoides* (NC_042932.1), *A. chocoense* (NC_042914.1), *Anemopaegma arvense* (NC_037228.1), *A. prostratum* (NC_042920.1), *Dolichandra cynanchoides* (NC_037460.1), *Neojobertia candolleana* (NC_036503.1), *Pleonotoma albiflora* (NC_037461.1), *Tanaecium tetragonolobum* (NC_027955.1), and *Tecomaria capensis* (NC_037462.1)) based on sequences obtained from the National Center for Biotechnology Information (NCBI). Sequences were aligned using MAFFT v7.313 (Katoh and Standley 2013) and the neighbor-joining (NJ) tree was constructed with RAxML v8.2.11 (Stamatakis 2014) to reveal the phylogenetic relationship of *C. speciosa*. The branch support was estimated with 1000 bootstrap replications (Figure 1). The cp genome will provide important data for the further studies of the genus *Catalpa* and of the family Bignoniaceae.

**Figure 1.**
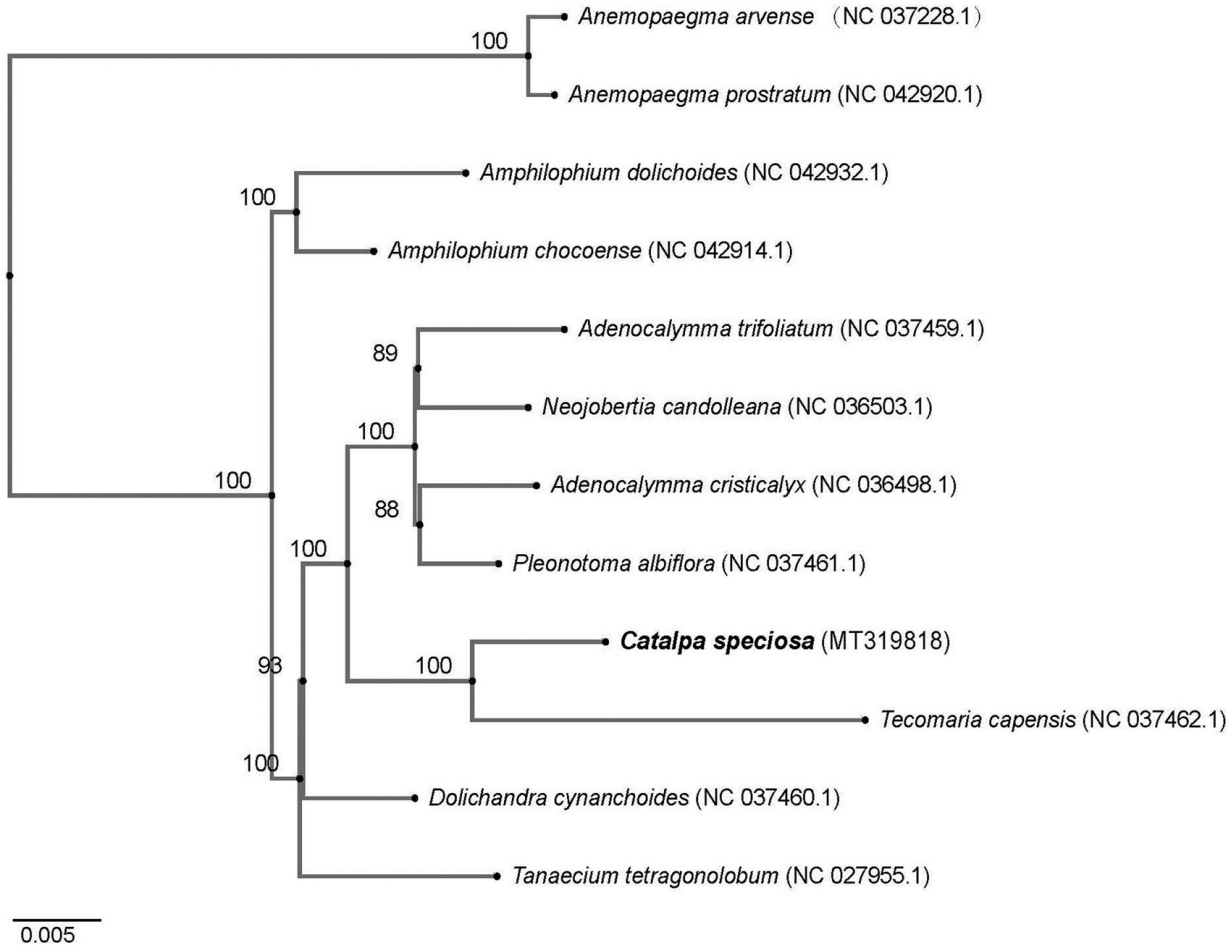
Phylogenetic tree inferred by the neighbor-joining (NJ) method from 12 complete chloroplast genomes. All the sequences were downloaded from NCBI GenBank.

## Data Availability

The data that support the findings of this study are openly available in GenBank (https://www.ncbi.nlm.nih.gov/genbank/) under accession number of MT319818.
